# Medical Household Waste as a Potential Environmental Hazard: An Ecological and Epidemiological Approach

**DOI:** 10.3390/ijerph20075366

**Published:** 2023-04-03

**Authors:** Adriana Benítez-Rico, Arizbeth Pérez-Martínez, Bryan Isaac Muñóz-López, Laura Martino-Roaro, Jorge Adan Alegría-Baños, Arely Vergara-Castañeda, Alejandro Islas-García

**Affiliations:** 1 Grupo de Investigación Desarrollo e Innovación en Ingeniería de Procesos y Nuevos Materiales, Vicerrectoría de Investigación, Universidad La Salle México, Mexico City 06140, Mexico; adriana.benitez@lasalle.mx; 2 Grupo de Investigación Desarrollo e Innovación en Ciencia y Tecnología Ambiental Aplicada, Vicerrectoría de Investigación, Universidad La Salle México, Mexico City 06140, Mexico; arizbeth.perez@lasalle.mx (A.P.-M.); alejandro.islas@lasalle.mx (A.I.-G.); 3 Programa de Maestría en Farmacología Clínica, Facultad de Ciencias Químicas, Universidad La Salle México, Mexico City 06140, Mexico; bi.ml@lasallistas.org.mx; 4 Grupo de Investigación Desarrollo e Innovación en Promoción y Educación para la Salud y Alimentación, Vicerrectoría de Investigación, Universidad La Salle México, Mexico City 06140, Mexico; jorge.alegria@lasallistas.org.mx; 5 Campus Ciudad de México, Centro Universitario Incarnate Word, Mexico City 03100, Mexico; lauramartinor@gmail.com; 6 Centro Oncológico Médica Sur, Mexico City 14050, Mexico

**Keywords:** medication disposal, drug disposal, pharmaceutical residues, emerging pollutants, environmental hazard, water bodies, wastewater, eco pharmacovigilance, Mexico

## Abstract

Nowadays, the contamination caused by emerging pollutants is a global concern due to the lack of scientific evidence to demonstrate the risk or toxicity to humans due to the presence of pharmaceutical residues in the environment. This study aimed to identify and describe the disposal practices of unused and unwanted medications, as well as to analyze and identify the most frequent drugs determined on water bodies adjacent to the biggest urban population in Mexico. A two-phase study with an epidemiological and an ecological assessment was performed. The epidemiological phase was carried out with a descriptive cross-sectional study among citizens from Mexico City and the metropolitan area using an electronic survey applied to 719 subjects aimed to assess practices in which pharmaceutical products are disposed. The ecological phase included a review of scientific reports. The results show that nearly 83.5% of those surveyed use inappropriate practices for disposal medicines, the main ones are through the municipal dump or directly in the drain. The ecological approach was carried out by a systematic literature review of original reports published between 2013 to 2023; information about the class of drugs, active substance, environmental compartments, location, and concentration was extracted and presented. Fifty-one different types of pharmaceutical residues were detected in wastewater in Mexico City in the last decade. The results of this study can contribute to the application of public policies for waste management authorities to mitigate the socio-environmental risks due to the inappropriate disposal of medicines.

## 1. Introduction

In recent years, there has been an increased international awareness on medical waste, including contaminated packaging, the improper disposal of unused or expired pharmaceuticals, as well as their possible associations with environmental and health effects [1].

One of the main reasons why medical drugs are found in the environment is because organisms does not absorb a certain percentage of medicines, or some metabolites are excreted through urine. Meanwhile, topical medications or medical personal care products are eliminated during showering. Besides, it has been suggested that disposal of unwanted medications in households made mostly through environmentally-unfavorable routes, such as rubbish bins, or a direct release into wastewater systems through the sink or toilet may contribute significantly to water and ground contamination, thus, forty-nine percent (49%) of generated pharmaceutical waste ends up in the local and surrounding areas, 21% contaminates the drainage system, and 25% is discharged into receiving waters [2,3].

In the case of Mexico City, the principal and biggest urban area in Mexico, some medical wastes, such as estrogens, ibuprofen, gemfibrozil, ketoprofen, diclofenac, salicylic acid, 2-ethylhexyl phthalate, buthylbencylphtalate, tryclosan, bysphenol A, and 4-nonilphenol, in the drinking water supply have been found since 2013 [4,5,6,7,8,9,10,11,12,13,14,15,16].

Even though there is no definitive scientific evidence to demonstrate the risk or toxicity to humans due the presence of pharmaceutical residues in the environment, it has been suggested that a negative or diffuse effect may result from an interaction between plants, animals, and humans, which can lead to chronic toxicity and cause genotoxicity, carcinogenicity, interference with hormone and immune systems, and drug resistant bacteria in the latter [17,18].

In this sense, the adverse effects of pharmaceutical effluents specifically on aquatic ecosystems are related to the exposure and bioaccumulation of these compounds in organisms, either through metabolic disorders, biochemical alteration, reproduction disruption, genotoxic effects, growth inhibition, behavioral changes, or mortality. These effects modify population dynamics and the trophic chain of ecosystems [19,20]. 

In response to this topic, at the government level, Mexico has standards to indicate the maximum permissible limits of contaminants in wastewater [21,22,23,24]. Expired medicines are considered as a hazardous waste [21], and in the specific case of medical wastes, The Official Mexican Standard NOM-073-ECOL-1996, published in 1996, establishes the maximum permissible limits of contaminants in wastewater discharges to water bodies from pharmaceutical and pharmachemical industries. This standard only considers five basic parameters to pharmaceutical wastes: pH, biochemical oxygen demand, chemical oxygen demand, fats and oils, and total suspended solids, and for the pharmachemical industry, adds cyanide concentrations [23]. 

Unfortunately, Mexican standards for wastewater do not consider specific compounds that, through the years, have been suggested to have a negative effect on the environment and human health. Furthermore, there are no regulations that establish the proper way to dispose of chemical and toxic waste as pharmaceutical residues, whether they are discarded by the industry or public population [13,14,15,16]. 

Instead, there are some strategies aimed to promote correct medication disposal, of which one stands out: the National Management System of Residuals of Containers and Medications (SINGREM), which is a non-profit civil organization created by the pharmaceutical industry and is supported by the Mexican health and environment authorities at the Federal and local level. It aims to responsibly address the problems generated by medications that expired in Mexican households, including the management and final disposal of expired medications and their surpluses in the homes of the user public, based on the General Law for prevention and management of waste [24,25]. 

This program is implemented on a national level to every community with over 100,000 inhabitants and their conurbation areas. Special containers are employed, in which the medicine user deposit expired or unused medications and a specialized recollection is programmed periodically. Finally, destruction takes place through authorized third parties, in compliance with the applicable Environment and Natural Resources Secretary (SEMARNAT) regulation, and the residuals undergo physical processes of trituration and then are co-processed in a cement furnace [24,25].

Considering that Mexico is a country with a high consumption of medications and that there are no studies regarding an integrative quantity assessment of the medical disposal in household wastes, nor the presence of pharmaceutical compounds in municipal garbage dumps, underground water, or surface water, we propose that assessing inadequate practices of the disposal of medicines could be related to the presence of drugs in the environment, and this evidence could contribute to reinforce the promotion of a correct unwanted medicines take-back, representing one of the few feasible solutions to reduce pharmaceutical discharges into the environment and, therefore, reduce adverse consequences for public health. Thus, this study was aimed to identify and describe the disposal practices of unused and unwanted medications, as well as to analyze and identify the most frequent drugs determined in water bodies adjacent to the biggest urban population in Mexico, Mexico City and the metropolitan area.

## 2. Materials and Methods

### 2.1. Study Design

This was a two-phase study in which epidemiological and ecological assessments are presented.

### 2.2. Epidemiological Approach

A descriptive cross-sectional study was performed among citizens from Mexico City and the metropolitan area, in which an electronic pretested survey was designed, validated, and used to collect data regarding risk practices related to the disposal of unused or expired medications. Inclusion criteria included subjects of either gender, aged between 18 to 75 years, living in Mexico City and the metropolitan area, including students, public and private sector employees, and the general population with a drug prescription for any acute disease in the last 12 months. Exclusion criteria considered those subjects under chronic pharmacological treatment, since regular use could suggest a bias to identify expired or unwanted drugs.

A sample size of 385 subjects was calculated considering a population of 9,209,944 residents of Mexico City with a confidence level of 95% and a sample error of 5%. Participants were invited using a convenience non-probability sampling.

An electronic survey consisted of three sections validated by experts. In the first one, the participant’s profile was assessed, including variables such as gender, age, job situation, work area, and consumption of medication practices, as well as the type of medication used in the last 12 months, generic or brand name. Participants were categorized into six different categories according to their pharmaceutical use: analgesic, antibiotics, non-steroidal anti-inflammatory drugs (NSAIDs), antacid, antihistamines, and skeletal muscle relaxants.

Section two describes the practices in which medications are stored into the household; this data was presented elsewhere. The third section of the survey, which will be analyzed and discussed at length in this article, describes the disposal practices of unwanted and unused medications. An open question was included to inquire how those surveyed dispose of the medications and to describe these practices. The rest of the questions focused on the general knowledge that the respondents have on the correct way to dispose of pharmaceutical products in specific containers, including where they have seen them and if they know what they are for.

### 2.3. Ecological Approach

This approach was made through an exhaustive review of scientific documents published in peer-reviewed journals during the period of 2013–2023 from databases such as Pubmed, EBSCO, Scopus, and Google Scholar, using key words such as “medicine”, “pharmaceutical”, “residues/pollution or emerging contaminants”, “water bodies”, sewage waters”, AND “Mexico City”, “Valley of México” “urban”. After an initial review, studies focused solely on the assessment and determination of the presence of a pharmaceutical residue in the natural environment, including surface waters, drinking waters, and wastewaters, and were contextualized within the current environmental and geographical framework. Regarding information of the type and quantity, if any, of medicines in water, ten studies were determined as relevant to the research and these articles were reviewed further. Information regarding type, category, and active substance of the medication, as well as environmental compartments, class, locality, and concentration if reported, was extracted and synthesized in this document in Appendix A.

In addition, a desk study reviewed the current national regulations in Mexico in which four national policies and guidelines relevant to this framework (environmental sanitation, water, and management of pharmaceutical waste) were reviewed. In addition, a bibliographic search was performed; once the documents were selected according to the scope and provided information, the variables of interest were obtained and categorized (type and concentration of pharmaceutical residues, if reported; specific region adjacent to the metropolitan area), and a matrix with environmental data was built.

### 2.4. Data Analysis

All returned surveys were double checked for accuracy and consistency by two blinded reviewers (LMR, AVC). The collected data was coded and then a single researcher categorized the answers collected on open questions. Analyses were performed using SPSS–IBM version 21^®^. The participants were classified into comparison groups according to gender and to the working field, as it has been suggested that these characteristics are considered confounding variables [26]. 

The nominal variables were described through absolute values and percentages and were compared between the groups using the χ^2^ test or Fisher’s exact test, while the continuous variables of the ratio were described using the mean and standard deviation. The continuous variables were tested for the normal distribution using the Kolmogorov–Smirnov test; if the variables did not meet the normality criterion, they were described as medians and 25 and 75 percentiles. The comparisons that were done to the independent variables and that marked the association were made using the Student’s t-test for independent samples or the Mann–Whitney test, as appropriate. A *p* value < 0.05 and *p* < 0.001 were considered statistically significant with 95% and 99% confidence, respectively.

For the environmental data assessment, 3130 papers were identified and reviewed, and only 10 of them were used to extract information about the presence and determination of drugs in the Valley of México. No statistical analyses were performed due the heterogeneity of the used methodologies and reporting of the findings. 

### 2.5. Ethical Considerations

Participation in this survey was voluntary and the identity of the respondents was kept confidential. Before answering the survey and after letting them know the purpose of the study, the respondents pre-approved its application with the question “Are you willing to participate?” Out of 747 people that were asked, 15 respondents denied the application of the survey. An ethics committee was not necessary because this study involved a risk lower than minimum and a secondary analysis of reports. The study was conducted according to the Mexican General Health Law and the International Organisms.

## 3. Results

### 3.1. Practices of Disposal of Unused, Unwanted, or Expired Medications

As can be observed in Table 1, a total of 719 surveys were analyzed, reaching a response rate of 96.2%; 58.6% of respondents were female, aged 33.6 ± 11.9 years old, while 41.3% of the surveyed were males, aged 31.9 ± 12.7 years old. Most of the subjects are full-time employees (46.6%), followed by students (26.4%) and half-time employees (10.4%), with unemployed being the less frequent answer (1.9%). Out of the 719 respondents, 92.8% claimed that they had used any type of medication in the last 12 months, 95.5% of these responses came from women and 88.9% from men. Thirty-nine different types of medications were reported, the most frequent being analgesics (62.4%), antibiotics (40.6%), non-steroidal anti-inflammatory drugs (NSAIDs) (30.7%), and antacids (21.0%). 

Several methods were reported for disposal of unused drugs; the most common was getting rid of the medication directly in the household trash (60.8%), followed by what respondents mentioned as containers (15.7%), meanwhile 7.5% of the respondents mentioned that they destroy or dilute the medication and 6.7% answered that they flush it down the toilet or drain.

**Figure 1:**
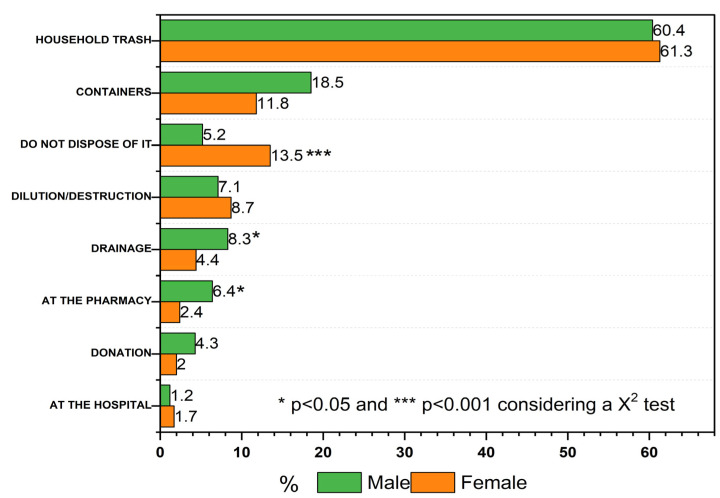
Disposal techniques according to gender.

As it can be seen in Figure 1, some differences were observed between genders for the following techniques: disposal in containers, drainage, and disposal at the pharmacy. According to the survey, 13.5% of males and 5.2% of females said that they do not dispose of their medications and instead they keep them. On the other hand, 8.3% of females surveyed stated that they dispose of medication via the drains, against 4.4% of male respondents, and lastly, 6.4% of female respondents said that they dispose of medications at the pharmacy, against 2.4% of male respondents. These differences implied statistical significance (*p* < 0.001).

According to the surveys, there are two main methods for final medication disposal: the first one is common household trash, and the second is the use of official medical containers. In addition, it was observed that depending on the work field, those surveyed use of one of these methods.

As it can be observed in Table 2, 26.4% of those surveyed work in some Health Sciences-related job, while 73.5% of them work in any other field. From the Health Sciences workers, 42.3% dispose of their medication in the household trash, while from the other worker group, 67.3% use the same disposal method. The containers method is used by 38.1% of the Health Sciences workers compared with the 7.8% of the other working group. 

As the current and main strategy for proper medication disposal, 34.5% of the respondents said they had seen these specific containers and only 27.3% mentioned that these are for the disposal of unused or expired medications. Meanwhile, the rest of those surveyed did not know the use of the containers or they are confused with some other waste collection points (batteries or electronic devices). These vessels were mostly seen in the pharmacy (24.6%), followed by hospitals (11%) and supermarkets (10.3%). To the group of respondents who work in a Health Sciences-related field, 43.9% use the containers found in pharmacies compared with the 17.8% surveyed from other work fields, followed by the 9.8% that use the containers found in supermarkets. 

Differences were observed regarding disposal methods between individuals that work in health-related areas versus individuals that have other jobs: Participants belonging to the latter group are more prone to dispose of the medications via the household trash (67.3%), while those working in health-related areas are more prone to dispose of medications via containers (38.1%) and at the Pharmacy (11.6%). 

Despite this difference, knowledge in the health-related areas of how and where they can dispose of the unused or expired medicines correctly did not even reach half of the positive answers.

### 3.2. Main Drugs Found in Water Bodies in Mexico City and Metropolitan Area

**Figure 2:**
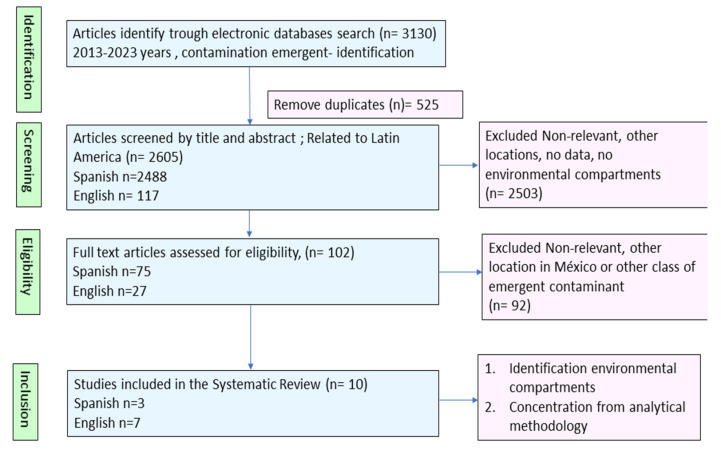
Disposal techniques according to gender.

In order to identify reported data of the presence of drugs and their quantification in the Valley of México, a systematic literature search was conducted using databases and a search strategy described in the methodology section. Criteria used to identify relevant published papers for the period between 2013 to 2023 was carried out in several steps, as in the flowchart detailed in Figure 2. The search was refined in English and Spanish, taking into account the language spoken in the study country.

After a review of titles, results that were irrelevant or repeated data were excluded. Only studies with two requirements were taken into account: those with the identification of drugs in environmental compartments and with concentration values that result from the quantification carried out by a developed and validated analytical methodology. Finally, information about class of drugs, active substance, environmental compartments, location, and concentration was extracted.

**Figure 3:**
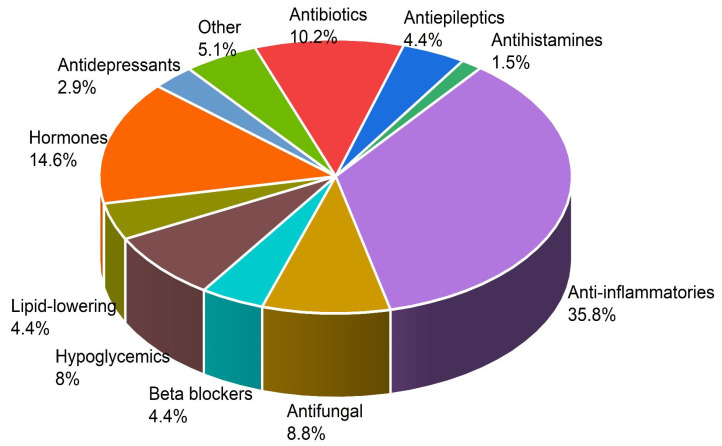
Percentages of different pollutants found in Mexico City and the metropolitan area (2013–2023) [14,15,16,27,28,29,30,31,32,33].

In total, 3130 papers were identified and reviewed, and only 10 of them were used to extract information about the presence and determination of drugs in the Valley of México. It is important to highlight that although the review covers until 2023, the most recent data reports are from 2019. In this research, 10 papers with 132 independent quantifications of drugs with different classes or active substances were detected, in which the presence of at least 51 different types of pharmaceutical residues were detected in wastewater in Mexico City (Appendix A). In addition, as can be seen in Figure 3, more than 50% of all the pharmaceutical wastes identified were anti-inflammatory, hormones, and antibiotics. These results are congruent, considering that in Mexico, drugs such as ibuprofen, paracetamol, and salicylic acid, among others, can be purchased without medical prescriptions.

The maximum concentrations detected for each type of drug in the different studies were as follows: anti-inflammatories (naproxen 7010 ng/L), hormones (androsterone 3020 ng/L), antibiotics (erythromycin 769 ng/L), antifungals (triclosan 988 ng/L), hypoglycemics (metformin 32,100 ng/L), others (teofiline 10,400 ng/L), antiepileptics (carbamazepine 678.3 ng/L), beta blockers (metoprolol 87.2 ng/L), lipid-lowering (clofibric acid 5856 ng/L), antidepressants (diazepam 2.61 ng/L), and antihistamines (acetaminophen 18,500 ng/L).

The presence of hormones in wastewater could be explained due to treatments used as hormone replacement therapy or fertility treatments, for the prescription of corticosteroids for autoimmune diseases, severe respiratory disorders, certain allergic conditions, and prescriptions to treat several dermatological conditions. All these compounds reach the water bodies when they are discarded in the urine or through expired medicines that are thrown directly down the drain. 

**Figure 4:**
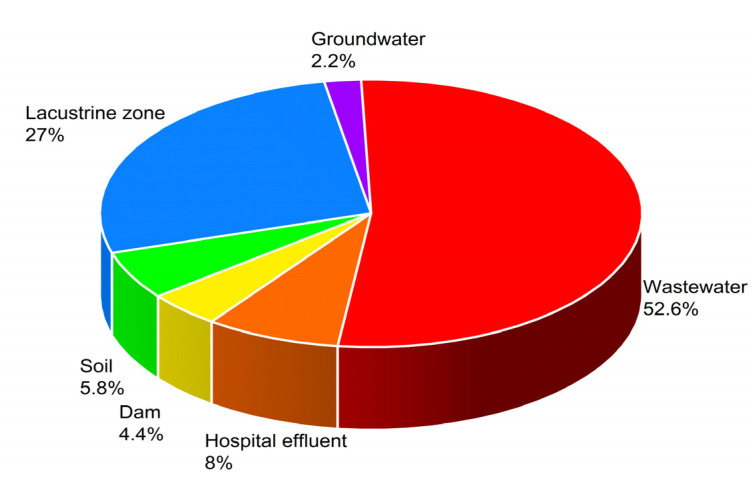
Distribution of water bodies with pollutants of pharmaceutical origin in Mexico City and the metropolitan area (2013–2023) [14,15,16,27,28,29,30,31,32,33].

According to the analyzed reports, domestic and industrial wastewater concentrates the largest amount of the identified medical drugs, followed by the lacustric zone of the region, which includes the south municipalities such as Xochimilco, Milpa Alta, and Tlahuac (Figure 4). 

## 4. Discussion

Mexico is a great market for drug manufacturers and medical devices, both from national and global companies, and nowadays is considered as the second largest pharmaceutical industry in Latin America. Hence, the presence of active pharmaceutical compounds, such as antibiotics, steroid hormones, antihypertensives, neuroactive drugs (antiepileptic and antidepressants), painkillers, or analgesic and anti-inflammatory drugs, have been determined in the environment, freshwater bodies, and in some cases, they are detected downstream of waste of water treatment plants or adjacent to fields receiving animal manures, representing a potential environmental hazard of exposure to these chemicals [14,15,16,27,28,29,30,31,32,33].

The results show that nearly 83.5% of those surveyed disposed of their pharmaceutical products in an incorrect way. The main method used to dispose of them is in the municipal dump, directly in the drain, or burning them, which is in accordance with some other reports [34,35,36,37,38], with very low awareness (10.5%) of them knowing how the medical drugs can be properly disposed, as reported previously [38,39].

Similar results were found in the study conducted by Zúñiga-Lemus et al. in Oaxaca, Mexico, where the surveyed population was not aware of how to properly dispose of medications and the unused medicines were kept at households for an average time of one year, disposing of them afterwards via the household trash or through the drainage [35], which is in contrast to that the observed in Sweden, where 85% of the respondents were aware that returning unused and expired medication to a pharmacy is the appropriate method for its disposal [40].

Although statistically significant differences according to the background of the participants were found, those who are working in a health-related area, as students or workers, showed a tendency to have better disposal practices, as seen in other reports [41,42]. There was a large proportion of participants presenting risk behaviors regarding the final management of medications, where 61.9% of those surveyed working in health-related areas do not dispose of medications appropriately, in spite of their scientific background, who should entail knowledge on this matter and, therefore, recognize the implications on the potential environmental and health hazards [41,42]. 

Similar reports have been made, in which pharmacists, residents, and medical students were assessed to identify the appropriate methods of medication disposal that they could use or recommend to patients, indicating that nearly 10–15% were able to recognize all the appropriate methods to discard medications [42]; almost two-thirds indicated complete lack of knowledge of documented guidelines for medication disposal in contrast with only 25% whom had specific training on disposal practices.

Antibiotics were the second most frequent discarded medication reported by the participants, and among the first ones found in water bodies adjacent to Mexico City. The information obtained is consistent with that reported in some urban areas in China [43,44], where a continual exposure to this kind of pollution could develop an antibacterial resistance in the natural environment, posing a direct risk for the population [43]. 

In addition, analgesics and NSAIDs were also mentioned as the most commonly discarded medications, as reported in other research [45,46], which have been world widely reported as one of the most dominant and frequently detected groups in environmental matrices including wastewater, surface water, suspended solids, sediments, groundwater, and even drinking water. There is definitive evidence for the adverse impacts of NSAID residues on scavenging birds and aquatic species [46].

The maximum reported values of drugs for Mexico City in wastewater, dam, groundwater, and the lacustrine zone do not exceed the critical environmental concentration (CEC) (Table 3). The CEC indicates an approximation of the concentration of the drug in the medium that can increase the concentration within an organism. For there to be acute toxicity of drugs at different levels of the trophic chain (phytoplankton, zooplankton, benthos, and fish), the concentrations must exceed 500,000 ng/L, while for chronic toxicity, the concentrations must be greater than 1000 ng/L [47]. Although the maximum values of the present study do not exceed these values, bioaccumulation in organisms can increase the concentrations of these compounds and generate adverse effects [48]. However, considering that most drugs have log Kow values between 2 and 5, it indicates that these compounds are potentially bioaccumulative in aquatic organisms [49]. In addition, the adsorption of drugs by microplastics has currently been demonstrated, which could increase the accumulation of these compounds within organisms in aquatic ecosystems [50]. On the other hand, there are reports that aquatic species of animals, plants, and bacteria react differently to various concentrations and exposure times, making it difficult to determine the large-scale effect of these pharmaceutical residues [51].

Furthermore, one of the biggest challenges for infection control worldwide and, therefore, a public health concern regarding environmental-gene interaction, is the bacterial resistance to *Enterococcus faecium, Escherichia coli, Sthapylococcus aureus, Klebsiella pneumoniae, Acinetobacter baumanni, Pseudomonas aeruginosa,* and some other Enterobacter species, which could lead to an increase in the morbidity and mortality rates [1,17].

In this sense, a study conducted by Guardabassi et al. demonstrated a possible increase in antibiotic-resistant bacteria in sewage associated with the discharge of wastewater from a pharmaceutical plant and a hospital using *Acinetobacter* species as environmental bacterial indicators [18]. The results indicated that the discharge of wastewater from the pharmaceutical plant was associated with an increase in the prevalence of both single and multiple antibiotic resistance among the *Acinetobacter* species in the sewers [2,18]. Besides, other data obtained suggests that metabolites of germifrozil, carbamazepine (carbamazepine epoxide), diclofenac (4′- and 5-hydroxy diclofenac), and atorvastatin (o- and p-hydroxy atorvastatin) are detected at higher concentrations than the parent pharmaceuticals into inflow proportional 24 h composite samples of wastewater effluent collected from urban Norwegian cities [52].

**Table 3:** Characteristics of the active substances registered for Mexico City associated with ecological effects.

Class of Drugs	Active Substance	Maximum Concentration (ng/L)	CEC (ng/L)	log Kow
Anti-inflammatories	Naproxen	7010	827,999	3.1
Hormones	Androsterone	3020	--	3.0
Antibiotics	Erythromycin	769	--	3.0
Antifungal	Triclosan	988	--	4.7
Hypoglycemics	Metformin	32,100	64,000,000	-2.6
Antiepileptics	Carbamazepine	678.3	346,496	2.2
Beta blockers	Metoprolol	87.2	15,390	1.7
Lipid-lowering	Clofibric acid	5856	--	2.5
Antidepressants	Diazepam	2.61	16,219	2.7
Antihistamines	Acetaminophen	18,500	24,000,000	0.3
Others	Teofiline	10,400	290,000,000	0.4

CEC = the predicted water concentrations that would elevate the plasma concentration in exposed fish. log Kow: Octanol-water partition coefficient. This table was prepared and compiled by authors based on reported data from [19,53,54].

As a response to this challenge, eco pharmacovigilance (EPV) [44,55,56] has arisen as an emerging science concerning the detection, assessment, understanding, and prevention of adverse effects related to the presence of pharmaceuticals in the environment, which affect human and other animals.

After the pharmaceutical compounds enter the environment, the active substances move from one environmental compartment to others, contaminating surface and ground water, soil, and even air. The highly liposoluble compounds can accumulate in fatty tissue of animals and return to food chain [57].

However, the possible negative effect to human beings is not as clear as for the environment; drinking water and food contain low levels of these chemical compounds but are not considered toxic to humans in the short term, but in long term, could be by its own toxic nature. Authors should discuss the results and how they can be interpreted from the perspective of previous studies and of the working hypotheses [58]. The findings and their implications should be discussed in the broadest context possible. Future research directions may also be highlighted.

## 5. Conclusions

This study shows that a large proportion of the general population, including those related to a health-field, do not dispose of medications appropriately and are not aware of the proper mechanisms to do it, even those who work in a health-related areas. Thus, one of the inputs of this study is the need to organize educational campaigns focused in the overall community considering integral strategies between industry, academia, and the government aimed to ensure that pharmaceuticals in the environment are managed appropriately in a timely way to prevent health risks.

On the other hand, we highlight the recognition of significant environmental issues associated with pharmaceuticals in the environment, as emerging contaminants and the need to increase the awareness for proper disposal, and whether the inadequate disposal of pharmaceuticals results in a direct impact on the environment and possibly on human health.

A parallel and integrative approach is required to perform more research focused on biological monitoring of different species, measurements, predictions, and the identification of potential effects of pharmaceutical pollutants to improve scientific understanding of pharmaceuticals in the environment. Additionally, one long-term response to this problem could be the development of a “green and sustainable pharmacy” as an effective measure to solve the issue of emerging pollution by pharmaceutical residues.

## Figures and Tables

**Table 1:** Characteristics of the sample of participants living in Mexico City and the metropolitan area according to gender.

Variable	Total n = 719	Female n = 422	Male n = 297	*p*
Age, years	32.9 ± 12.2	33.6 ± 11.9	31.9 ± 12.7	0.090
Occupation				
Full-time employee	46.6 (335)	41.7 (176)	53.5 (159)	<0.001 ***
Student	26.4 (190)	23 (97)	31.3 (93)	
Half-time employee	10.4 (75)	41.7 (176)	53.5 (159)	
Housewives/home staying	9.3 (67)	15.6 (66)	0.3 (1)	
Others	2.8 (20)	3.3 (14)	2 (6)	
Retired	2.5 (18)	2.4 (10)	2.7 (8)	
Unemployed	1.9 (14)	2.4 (10)	1.3 (4)	
Use of medication in the last year	92.8 (667)	95.5 (405)	88.9 (264)	<0.001 ***
Type of medication				
Analgesic	62.4 (449)	66.8 (282)	56.2 (167)	<0.05 *
Antibiotic	40.6 (292)	44.3 (187)	35 (104)	
NSAIDs	30.7 (221)	31.5 (133)	29.6 (88)	
Antacid	21 (151)	22 (93)	19.5 (58)	
Antihistamines	11.9 (86)	15.9 (67)	6.4 (19)	
Skeletal muscle relaxants	10.6 (76)	10.9 (46)	10.1 (3)	

NSAIDs: non-steroidal anti-inflammatory drugs. Data is presented as n (%) or mean ± standard deviation, depending on the value of the variable. * *p* < 0.05 and *** *p* < 0.001 considering a *X*^2^ test.

**Table 2:** Disposal techniques and knowledge of specific containers for the proper handling of medication, according to employment in the health-related area.

	Total n = 719	Health Sciences-Related n = 190	Not Health Sciences-Related n = 529	*p*
How do you dispose of medications?				
Household trash	60.7 (436)	42.3 (80)	67.3 (356)	<0.001 ***
Containers	15.7 (113)	38.1 (72)	7.8 (41)	<0.001 ***
Do not dispose of it	8.6 (62)	6.9 (13)	9.3 (49)	0.367
Destruction/Dilution	7.5 (54)	4.8 (9)	8.5 (45)	0.108
Drainage	6.7 (48)	7.9 (15)	6.2 (33)	0.402
Pharmacy	4.7 (34)	11.6 (22)	2.3 (12)	<0.001 ***
Donation	3.3 (24)	2.6 (5)	3.6 (19)	0.643
Hospital	1.4 (10)	0 (0)	1.9 (10)	0.071
Has seen the containers according to employment in Health-related area				
Has not seen the containers	65.5 (470)	43.4 (82)	73.3 (388)	<0.001 ***
Does not know what the containers are for	72.7 (522)	51.9 (98)	80.2 (424)	<0.001 ***
Where the subjects have seen the containers according to employment in Health-related area				
Pharmacies	24.7 (177)	43.9 (83)	17.8 (94)	<0.001 ***
Hospitals	11 (79)	20.1 (38)	7.8 (41)	<0.001 ***
Supermarkets	10.3 (74)	13.8 (26)	9.1 (48)	0.72
Lab Testing Provider	2.5 (18)	2.1 (4)	2.6 (14)	0.793
Others	1.8 (13)	5.8 (11)	0.4 (2)	<0.001 ***
Schools	1.1 (8)	2.1 (4)	0.8 (4)	0.217

Data is presented as n (%) or mean ± standard deviation, depending on the value of the variable. *** *p* < 0.001 considering a *X*^2^ test.

## Data Availability

All data are available in the manuscript when requested to the corresponding author.

## Appendix A

The Table A1 presents the ecological data obtained of published reports.

**Table A1:** Distribution of pharmaceutical residues in adjacent water bodies to Mexico City and the metropolitan area (2013–2023).

Class of Drugs	Active Substance	Environmental Compartments	Location	Concentration	Author, Year.
Antibiotics	Ciprofloxacine	Wastewater	Metropolitan area-Estado de México	315–373 ng/L	Estrada-Arriaga, et al. (2016) [30]
	Claritromicine	Wastewater	Metropolitan area-Estado de México	39.8–567 ng/L	Estrada-Arriaga, et al. (2016) [30]
	Eritromicine	Wastewater	Metropolitan area-Estado de México	200–769 ng/L	Estrada-Arriaga, et al. (2016) [30]
	Lincomicine	Wastewater	Metropolitan area-Estado de México	33.5–550 ng/L	Estrada-Arriaga, et al. (2016) [30]
	Ofloxacine	Wastewater	Metropolitan area-Estado de México	51–107 ng/L	Estrada-Arriaga, et al. (2016) [30]
	Oxitetracicline	Wastewater	Metropolitan area-Estado de México	31.7 ng/L	Estrada-Arriaga, et al. (2016) [30]
	Penicilic V	Hospital effluent	Metropolitan area-Estado de México	<LOQ	Pérez- Alvarez, et al. (2018) [33]
	Penicilin G	Hospital effluent	Metropolitan area-Estado de México	<LOQ	Pérez- Alvarez, et al. (2018) [33]
	Penicilin G	Dam	Metropolitan area-Estado de México	249–280 ng/L	Pérez-Coyotl I., et al. (2019) [16]
	Penicilin V	Dam	Metropolitan area-Estado de México	11.49–14.34 ng/L	Pérez-Coyotl I., et al. (2019) [16]
	Sulfadiazine	Wastewater	Metropolitan area-Estado de México	62.9–100 ng/L	Estrada-Arriaga, et al. (2016) [30]
	Sulfametoxazole	Wastewater	Metropolitan area-Estado de México	279–641 ng/L	Estrada-Arriaga, et al. (2016) [30]
	Triclosan	Lacustrine zone	Xochimilco, México City	<LOQ	Hernández-Quiroz, et al. (2019) [32]
	Trimethoprim	Wastewater	Metropolitan area-Estado de México	220–601 ng/L	Estrada-Arriaga, et al. (2016) [30]
Antidrepressants	Amitriptyline	Wastewater	Metropolitan area-Estado de México	0.693–0.942 ng/L	Estrada-Arriaga, et al. (2016) [30]
	Amitriptyline	Wastewater	Metropolitan area-Estado de México	1.3 ng/L	Estrada-Arriaga, et al. (2016) [30]
	Diazepam	Wastewater	Metropolitan area-Estado de México	2.16–2.61 ng/L	Estrada-Arriaga, et al. (2016) [30]
	Meprobamate	Wastewater	Metropolitan area-Estado de México	<LOQ	Estrada-Arriaga, et al. (2016) [30]
Antiepileptics	Carbamazepine	Wastewater	México City	37.4–43.7 ng/L	Estrada-Arriaga, et al. (2016) [30]
	Carbamazepine	Wastewater	Coyoacán, México City	125–162 ng/L	Melo-Guimarães, et al. (2013) [27]
	Carbamazepine	Soil	Xochimilco, México City	17.2–23.6 ng/g	Campos, S. et al. (2017) [31]
	Carbamazepine	Lacustrine zone	Xochimilco, Agricole Zone, México City	40.8–678.3 ng/L	Campos, S. et al. (2017) [31]
	Carbamazepine	Lacustrine zone	Xochimilco, Turistic Zone, México City	29.2–54.5 ng/L	Campos, S. et al. (2017) [31]
	Carbamazepine	Lacustrine zone	Xochimilco, Urban Zone, México City	20.0–78.3 ng/L	Campos, S. et al. (2017) [31]
Antifungal	Clorophene	Wastewater	Coyoacán, México City	Inffluent 0.23–4.60 ng/L Efluent 0.95–1.38 ng/L	Peña-Alvarez A., et al. (2015) [29]
	Clorophene	Wastewater	Coyoacán, México City	Inffluent 0.07–0.21 ng/L	Peña-Alvarez A., et al. (2015) [29]
	Clorophene	Wastewater	Iztapalapa, México City	Inffluent 0.14–5.04 ng/L Efluent 0.25–1.34 ng/L	Peña-Alvarez A., et al. (2015) [29]
	Clorophene	Wastewater	Iztapalapa, México City		Peña-Alvarez A., et al. (2015) [29]
	Triclosan	Groundwater	Gustavo A. Madero, México City	1–345 ng/L	Felix-Cañedo, et al. (2013) [15]
	Triclosan	Wastewater	Coyoacán, México City	801–988 ng/L	Melo-Guimarães, et al. (2013) [27]
	Triclosan	Wastewater	Coyoacán, México City	Inffluent 2.50–9.34 ng/L	Peña-Alvarez A., et al. (2015) [29]
	Triclosan	Wastewater	Coyoacán, México City	Efluent 0.32–2.63 ng/L	Peña-Alvarez A., et al. (2015) [29]
	Triclosan	Wastewater	Coyoacán, México City	Inffluent 0.91–1.59 ng/L	Peña-Alvarez A., et al. (2015) [29]
	Triclosan	Wastewater	Iztapalapa, México City	Efluent 0.08–7.33 ng/L	Peña-Alvarez A., et al. (2015) [29]
	Triclosan	Wastewater	Metropolitan area-Estado de México	531–383 ng/L	Estrada-Arriaga, et al. (2016) [30]
	Triclosan	Dam	Metropolitan area-Estado de México	16–19 ng/L	Felix-Cañedo, et al. (2013) [15]
	Triclosan	Lacustrine zone	Xochimilco, México City	26.41–71.93 ng/μL	Díaz-Torres, et al. (2013) [28]
	Triclosan	Wastewater	Metropolitan area-Estado de México	197–287 ng/L	Peña-Alvarez A., et al. (2015) [29]
	Triclosan	Lacustrine zone	Xochimilco, México City	<LOD	Hernández-Quiroz, et al.(2019) [32]
Analgesic	Acetominophene	Wastewater	Metropolitan area-Estado de México	6940–18,500 ng/L	Estrada-Arriaga, et al. (2016) [30]
	Acetaminophen	Hospital effluent	Metropolitan area-Estado de México	2.66 µg/L	Peña-Alvarez A., et al. (2015) [29]
	Acetaminophen	Dam	Metropolitan area-Estado de México	1124–9156 ng/L	Pérez-Coyotl I., et al. (2018) [16]
	Diclofenac	Groundwater	Gustavo A. Madero, México City	1 ng/L	Felix-Cañedo, et al. (2013) [15]
	Diclofenac	Wastewater	Coyoacán, México City	2.327–3.043 ng/L	Melo-Guimarães, et al. (2013) [15]
NSADs	Diclofenac	Hospital effluent	Metropolitan area-Estado de México	0.59 µg/L	Pérez- Alvarez, et al. (2018) [33]
	Diclofenac	Dam	Metropolitan area-Estado de México	28–32 ng/L	Felix-Cañedo, et al. (2013) [15]
	Diclofenac	Soil	Xochimilco, México City	<LOQ	Campos, S. et al. (2017) [31]
	Diclofenac	Lacustrine zone	Xochimilco, Agricole Zone, México City	2161.5 ng/L	Campos, S. et al. (2017) [31]
	Diclofenac	Lacustrine zone	Xochimilco, Turistic Zone, México City	419.5 ng/L	Campos, S. et al. (2017) [31]
	Diclofenac	Lacustrine zone	Xochimilco, Urban Zone, México City	586.5 ng/L	Campos, S. et al. (2017) [31]
	Ibuprophene	Wastewater	Coyoacán, México City	Inffluent 0.41–2.81 ng/L Efluent 0.02–0.16 ng/L	Pérez- Alvarez, et al. (2018) [33]
	Ibuprophene	Wastewater	Coyoacán, México City	Inffluent 0.23–0.87 ng/L	Peña-Alvarez A., et al. (2015) [29]
	Ibuprophene	Wastewater	Iztapalapa, México City	Inffluent 0.38–28.9 ng/L Efluent 0.06–0.34 ng/L	Peña-Alvarez A., et al. (2015) [29]
	Ibuprophene	Wastewater	Iztapalapa, México City		Peña-Alvarez A., et al. (2015) [29]
	Ibuprophene	Wastewater	Metropolitan area-Estado de México	1620–5410 ng/L	Estrada-Arriaga, et al. (2016) [30]
	Ibuprophene	Wastewater	Metropolitan area-Estado de México	404–2140 ng/L	Estrada-Arriaga, et al. (2016) [30]
	Ibuprophene	Wastewater	Coyoacán, México City	561–884 ng/L	Melo-Guimarães, et al. (2013) [27]
	Ibuprophene	Hospital effluent	Metropolitan area-Estado de México	0.62 µg/L	Pérez- Alvarez, et al. (2018) [33]
	Ibuprophene	Soil	Xochimilco, México City	144.0–407.9 ng/g	Campos, S. et al. (2017) [31]
	Ibuprophene	Lacustrine zone	Xochimilco, México City	0.5–13.9 μg/L	Hernández-Quiroz, et al. 2019) [32]
	Ibuprophene	Lacustrine zone	Xochimilco, Agricole Zone, México City	4943.6 ng/L	Campos, S. et al. (2017) [31]
	Ibuprophene	Lacustrine zone	Xochimilco, Turistic Zone, México City	2179.5 ng/L	Campos, S. et al. (2017) [31]
	Ibuprophene	Lacustrine zone	Xochimilco, Urban Zone, México City	2803.2 ng/L	Campos, S. et al. (2017) [31]
	Ketoprophene	Wastewater	Coyoacán, México City	170–266 ng/L	Melo-Guimarães, et al. (2013) [27]
	Ketoprophene	Dam	Metropolitan area-Estado de México	21–42 ng/L	Felix-Cañedo, et al. (2013) [15]
	Ketoprophene	Soil	Xochimilco, México City	182.3 ng/g	Campos, S. et al. (2017) [31]
	Ketoprophene	Lacustrine zone	Xochimilco, Agricole Zone, México City	4179.1 ng/L	Campos, S. et al. (2017) [31]
	Ketoprophene	Lacustrine zone	Xochimilco, Turistic Zone, México City	466.0 ng/L	Campos, S. et al. (2017) [31]
	Ketoprophene	Lacustrine zone	Xochimilco, Urban Zone, México City	619.3 ng/L	Campos, S. et al. (2017) [31]
	Naproxen	Wastewater	Coyoacán, México City	Inffluent 54.36–3.45 ng/L Effluent 0.41 ng/L	Peña-Alvarez A., et al. (2015) [29]
	Naproxen	Wastewater	Coyoacán, México City	Inffluent 2.85–20.34 ng/L	Peña-Alvarez A., et al. (2015) [29]
	Naproxen	Wastewater	Iztapalapa, México City	Inffluent 3.47–51.13 ng/L Effluent 0.20–0.76 ng/L	Peña-Alvarez A., et al. (2015) [29]
	Naproxen	Wastewater	Iztapalapa, México City		Peña-Alvarez A., et al. (2015) [29]
	Naproxen	Wastewater	Metropolitan area-Estado de México	2090–7010 ng/L	Estrada-Arriaga, et al. (2016) [30]
	Naproxen	Wastewater	Coyoacán, México City	16.633–18.466 ng/L	Melo-Guimarães, et al. (2013) [27]
	Naproxen	Hospital effluent	Metropolitan area-Estado de México	1.79 µg/L	Pérez- Alvarez, et al. (2018) [33]
	Naproxen	Dam	Metropolitan area-Estado de México	8.5 ng/L	
	Naproxen	Dam	Metropolitan area-Estado de México	52–186 ng/L	Felix-Cañedo, et al. (2013) [15]
	Naproxen	Soil	Xochimilco, México City	113.4 ng/g	Campos, S. et al. (2017) [31]
	Naproxen	Lacustrine zone	Xochimilco, Agricole Zone, México City	<LOD (50 ng/L)	Campos, S. et al. (2017) [31]
	Naproxen	Lacustrine zone	Xochimilco, Turistic Zone, México City	<LOD (50 ng/L)	Campos, S. et al. (2017) [31]
	Naproxen	Lacustrine zone	Xochimilco, Urban Zone, México City	<LOD (50 ng/L)	Campos, S. et al. (2017) [31]
	Salicylic acid	Groundwater	Gustavo A. Madero, México City	1–464 ng/L	Felix-Cañedo, et al. (2013) [15]
	Salicylic acid	Wastewater	Coyoacán, México City	23.223–28.478 ng/L	Melo-Guimarães, et al. (2013) [27]
	Salicylic acid	Dam	Metropolitan area-Estado de México	29–309 ng/L	Felix-Cañedo, et al. (2013) [15]
	Salicylic acid	Soil	Xochimilco, México City	249.1 ng/g	Campos, S. et al. (2017) [31]
	Salicylic acid	Lacustrine zone	Xochimilco, Agricole Zone, México City	943.5 ng/L	Campos, S. et al. (2017) [31]
	Salicylic acid	Lacustrine zone	Xochimilco, Turistic Zone, México City	841.0 ng/L	Campos, S. et al. (2017) [31]
	Salicylic acid	Lacustrine zone	Xochimilco, Urban Zone, México City	339.9 ng/L	Campos, S. et al. (2017) [31]
Beta blockers	Atenolol	Wastewater	Metropolitan area-Estado de México	38.2–69.3 ng/L	Estrada-Arriaga, et al. (2016) [30]
	Atenolol	Hospital effluent	Metropolitan area-Estado de México	<LOQ	Pérez- Alvarez, et al. (2018) [33]
	Deshidronifedipine	Wastewater	Metropolitan area-Estado de México	<LOQ	Estrada-Arriaga, et al. (2016) [30]
	Metroprolol	Wastewater	Metropolitan area-Estado de México	25.9–87.2 ng/L	Estrada-Arriaga, et al. (2016) [30]
	Metroprolol	Hospital effluent	Metropolitan area-Estado de México	<LOQ	Pérez- Alvarez, et al. (2018) [33]
	Valsartan	Wastewater	Metropolitan area-Estado de México	36.1–85.6 ng/L	Estrada-Arriaga, et al. (2016) [30]
Hormones	17α-etinilestradiol	Wastewater	Coyoacán, México City	34–41 ng/L	Melo-Guimarães, et al. (2013) [27]
	17α-etinilestradiol	Wastewater	Gustavo A. Madero, México City	4–93 ng/L	Estrada-Arriaga, et al. (2016) [30]
	17β-estradiol	Wastewater	Metropolitan area-Estado de México	44.8 ng/L	Estrada-Arriaga, et al. (2016) [30]
	17β-estradiol	Hospital effluent	Metropolitan area-Estado de México	0.08 µg/L	Pérez- Alvarez, et al. (2018) [33]
	Androstenedione	Wastewater	Metropolitan area-Estado de México	145–371 ng/L	Estrada-Arriaga, et al. (2016) [30]
	Androsterone	Wastewater	Metropolitan area-Estado de México	1980–3020 ng/L	Estrada-Arriaga, et al. (2016) [30]
	B-estradiol	Wastewater	Coyoacán, México City	13–14 ng/L	Melo-Guimarães, et al. (2013) [27]
	B-estradiol	Lacustrine zone	Xochimilco, México City	<LOD	Hernández-Quiroz, et al.(2019) [32]
	Estradiol	Wastewater	Gustavo A. Madero, México City	4-93 ng/L	Estrada-Arriaga, et al. (2013) [14]
	Estradiol	Lacustrine zone	Xochimilco, México City	0.24–1.72 ng/μL	Díaz-Torres, et al. (2013) [28]
	Estrone	Wastewater	Coyoacán, México City	22–25 ng/L	Melo-Guimarães, et al. (2013) [27]
	Estrone	Wastewater	Gustavo A. Madero, México City	4-93 ng/L	Estrada-Arriaga, et al. (2013) [13]
	Estrone	Lacustrine zone	Xochimilco, México City	1.02–10.38 ng/μL	Díaz-Torres, et al. (2013) [28]
	Ibuprophene	Dam	Metropolitan area-Estado de México	15–45 ng/L	Felix-Cañedo, et al. (2013) [15]
	Estrone	Wastewater	Metropolitan area-Estado de México	35–65.4 ng/L	Estrada-Arriaga, et al. (2016) [30]
	Mestranol	Wastewater	Metropolitan area-Estado de México	947 ng/L	Estrada-Arriaga, et al. (2016) [30]
	Progesterone	Wastewater	Metropolitan area-Estado de México	26–47.4 ng/L	Estrada-Arriaga, et al. (2016) [30]
	Testosterone	Wastewater	Metropolitan area-Estado de México	24.9–79.3 ng/L	Estrada-Arriaga, et al. (2016) [30]
	17α-etinilestradiol	Wastewater	Metropolitan area-Estado de México	<LOD (8 ng/L)	Estrada-Arriaga, et al. (2013) [13]
	Estradiol	Wastewater	Metropolitan area-Estado de México	12–27 ng/L	Estrada-Arriaga, et al. (2013) [13]
	Estrona	Wastewater	Metropolitan area-Estado de México	14–54 ng/L	Estrada-Arriaga, et al. (2013) [13]
Hyperglycemic	Glibenclamide	Hospital effluent	Metropolitan area-Estado de México		Pérez- Alvarez, et al. (2018) [33]
	Glibenclamide	Dam	Metropolitan area-Estado de México	353–3449 ng/L	Pérez-Coyotl I., et al. (2019) [16]
	Metformin	Hospital effluent	Metropolitan area-Estado de México		Pérez- Alvarez, et al. (2018) [33]
	Metformin	Dam	Metropolitan area-Estado de México	378–11,694 ng/L	Pérez-Coyotl I., et al. (2019) [16]
	Glibenclamide	Wastewater	Metropolitan area-Estado de México	23.6–22.8 ng/L	Estrada-Arriaga, et al. (2016) [30]
	Metformin	Wastewater	Metropolitan area-Estado de México	13,400–32,100 ng/L	Estrada-Arriaga, et al. (2016) [30]
Lypid lowering	Clofibric acid	Soil	Xochimilco, México City	118.6–203.2 ng/g	Campos, S. et al. (2017) [31]
	Clofibric acid	Lacustrine zone	Xochimilco, Agricole Zone, México City	1495.9 ng/L	Campos, S. et al. (2017) [31]
	Clofibric acid	Lacustrine zone	Xochimilco, Turistic Zone, México City	2737.8–5856 ng/L	Campos, S. et al. (2017) [31]
	Clofibric acid	Lacustrine zone	Xochimilco, Urban Zone, México City	402.2 ng/L	Campos, S. et al. (2017) [31]
	Gemfibrozil	Wastewater	Metropolitan area-Estado de México	276–605 ng/L	Estrada-Arriaga, et al. (2016) [30]
	Gemfibrozil	Dam	Metropolitan area-Estado de México	9–10 ng/L	Felix-Cañedo, et al. (2013) [15]
	Gemfibrozil	Soil	Xochimilco, México City	37.2 ng/g	Campos, S. et al. (2017) [31]
	Gemfibrozil	Lacustrine zone	Xochimilco, Agricole Zone, México City	<LOQ (50 ng/L)	Campos, S. et al. (2017) [31]
	Gemfibrozil	Lacustrine zone	Xochimilco, Turistic Zone, México City	500.1 ng/L	Campos, S. et al. (2017) [31]
	Gemfibrozil	Lacustrine zone	Xochimilco, Urban Zone, México City	707.9 ng/L	Campos, S. et al. (2017) [31]
	Gemfibrozil	Wastewater	Coyoacán, México City	2.103–3.696 ng/L	Melo-Guimarães, et al. (2013) [27]
	Atorvastatine	Wastewater	Metropolitan area-Estado de México	9.1–9.93 ng/L	Estrada-Arriaga, et al. (2016) [30]
	Albuterol	Wastewater	Metropolitan area-Estado de México	6.29–11.5 ng/L	Estrada-Arriaga, et al. (2016) [30]
	Amphetamine	Wastewater	Metropolitan area-Estado de México	16.4–46.1 ng/L	Estrada-Arriaga, et al. (2016) [30]
	Androsterone	Wastewater	Metropolitan area-Estado de México	122–189 ng/L	Estrada-Arriaga, et al. (2016) [30]
Other	Cafeine	Wastewater	Metropolitan area-Estado de México	4920–6430 ng/L	Estrada-Arriaga, et al. (2016) [30]
	Enalapril	Wastewater	Metropolitan area-Estado de México	22–42.5 ng/L	Estrada-Arriaga, et al. (2016) [30]
	Teofiline	Wastewater	Metropolitan area-Estado de México	3900–10,400 ng/L	Estrada-Arriaga, et al. (2016) [30]
	Theobromine	Wastewater	Metropolitan area-Estado de México	2870–8360 ng/L	Estrada-Arriaga, et al. (2016) [30]
	Difenhidramine	Wastewater	Metropolitan area-Estado de México	28.2 ng/L	Estrada-Arriaga, et al. (2016) [30]

NSADs; non-steroidal anti-inflammatory drugs, <LOQ;Limit of quantification, <LOD; Limit of detection.
